# Bromophenol Bis (2,3,6-Tribromo-4,5-dihydroxybenzyl) Ether Protects HaCaT Skin Cells from Oxidative Damage via Nrf2-Mediated Pathways

**DOI:** 10.3390/antiox10091436

**Published:** 2021-09-09

**Authors:** Hui Dong, Mingfei Liu, Li Wang, Yankai Liu, Xuxiu Lu, Dimitrios Stagos, Xiukun Lin, Ming Liu

**Affiliations:** 1 Key Laboratory of Marine Drugs, Ministry of Education, School of Medicine and Pharmacy, Ocean University of China, Qingdao 266003, China; donghui01997@163.com (H.D.); 21200831185@stu.ouc.edu.cn (M.L.); wl1263467376@163.com (L.W.); liuyankai@ouc.edu.cn (Y.L.); xuxiulu@126.com (X.L.); 2 Laboratory for Marine Drugs and Bioproducts of Qingdao National Laboratory for Marine Science and Technology, Qingdao 266237, China; 3 Department of Biochemistry and Biotechnology, School of Health Sciences, University of Thessaly, Biopolis, 41500 Larissa, Greece; stagkos@med.uth.gr; 4 Department of Pharmacology, School of Pharmacy, Southwest Medical University, 319 Zhongshan Road, Jiangyang, Luzhou 646000, China; xiukunlin@126.com

**Keywords:** bis (2,3,6-tribromo-4,5-dihydroxybenzyl) ether, HaCaT, Nrf2, ROS, oxidative stress

## Abstract

Excessive reactive oxygen species (ROS) promotes the oxidative stress of keratinocytes, eventually causing cell damage. The natural bromophenol bis (2,3,6-tribromo-4,5-dihydroxybenzyl) ether (BTDE) from marine red algae has been reported to have a varied bioactivity; however, its antioxidant effect has yet to be investigated systemically. Our present work aimed to explore the antioxidant effect of BTDE both on the molecular and cellular models and also to illustrate the antioxidant mechanisms. Our results showed that BTDE could effectively scavenge ABTS free radicals and protect HaCaT cells from damage induced by H_2_O_2_. Mechanism studies in HaCaT cells demonstrated that BTDE attenuated hydrogen peroxide (H_2_O_2_)-induced ROS production, reduced the malondialdehyde (MDA) level, decreased the oxidized glutathione (GSSG)/glutathione (GSH) ratio, and increased the antioxidant enzyme superoxide dismutase (SOD). Moreover, BTDE could inhibit the expression of Kelch-like epichlorohydrin-associated protein 1 (Keap1) and increase the expression of both nuclear factor erythroid 2-related factor 2 (Nrf2) and its downstream proteins TrXR1, HO-1, and NQO1. BTDE also activated the upstream signaling pathway of Nrf2 such as AKT pathway, while not activating the ERK or AMPKα pathways. In general, BTDE is a promising antioxidant to protect HaCaT cells against oxidative damage via Nrf2-mediated pathways.

## 1. Introduction

Reactive oxygen species (ROS) are produced in the process of physiologic activity and are necessary for the basic biological processes of keratinocytes, such as proliferation and differentiation [1]. However, the excessive production of cellular ROS may attenuate the skin’s antioxidant system and cause oxidative damage, atopic dermatitis, premature skin aging, skin cancer, and other diseases [2]. Up to now, there are no clinical drugs for the treatment of skin oxidative damage. It is believed that both exogenous and endogenous oxidative stress lead to the excessive production of ROS in keratinocytes. Therefore, the balance between the ROS production and its elimination maintains the skin redox balance [3,4,5]. Consequently, removal of excessive ROS and stimulating cellular response to ROS have positive significance for the treatment of the aforementioned skin diseases. Therefore, agents that can inhibit ROS production and/or stimulate cellular response to eliminate the high levels of ROS exert a vital role in confronting oxidative stress of skin cells.

Studies have revealed that nuclear factor erythroid 2-related factor 2 (Nrf2) can be considered one of the most important molecules in the cellular response to oxidative stress [6]. Normally, Nrf2 is regulated by its negative regulator Kelch-like epichlorohydrin-associated protein 1 (Keap1) via the ubiquitin proteasome system [7]. Under oxidative stress, Nrf2 dissociates from Keap1 and subsequently translocates to the nucleus, where Nrf2 binds to the antioxidant response elements (ARE) and activates ARE-driven genes encoding antioxidant and cytoprotective enzymes, including heme oxygenase-1 (HO-1), NAD(P)H: quinone oxidoreductase 1 (NQO1), superoxide dismutase (SOD), and thioredoxin reductase (TrxR) [8,9]. In addition, Nrf2 is also the central signal switch of some antioxidant enzymes involved in the synthesis and metabolism of glutathione, such as glutamate-cysteine ligase and glutathione transferase [10]. It is a feasible and promising strategy to modulate the Nrf2 pathway by using small compounds in order to overcome oxidative damage. For example, docosahexaenoic acid exerted antioxidant effects via inducing Keap1 degradation, thereby preventing Keap1-mediated Nrf2 inactivation [11]. Moreover, licochalcone A could counteract the oxidative stress on skin by up-regulating Nrf2 expression [12], and andrographolide sodium bisulfate could reduce ultraviolet-induced oxidative damage in HaCaT cells by activating the Keap1/Nrf2 pathway [13].

Bromophenols (BPs) are important secondary metabolites in marine resources and have a variety of biological activities [14,15,16]. Recent studies have shown that some BPs could modulate the Keap1/Nrf2 pathway and therefore possess promising antioxidant activity. For example, 3-bromo-4,5-dihydroxybenzaldehyde exhibited excellent antioxidant activity by regulating Nrf2 and its downstream proteins [17,18], while synthesized nitrogen-containing heterocycle BP 1-(4-(4-bromo-2-(2-bromo-4,5-dihydroxybenzoyl)benzyl)piperazin-1-yl) could effectively activate Nrf2, triggering downstream cytoprotective genes by targeting the Nrf2-Keap1 interaction [19].

**Figure 1:**
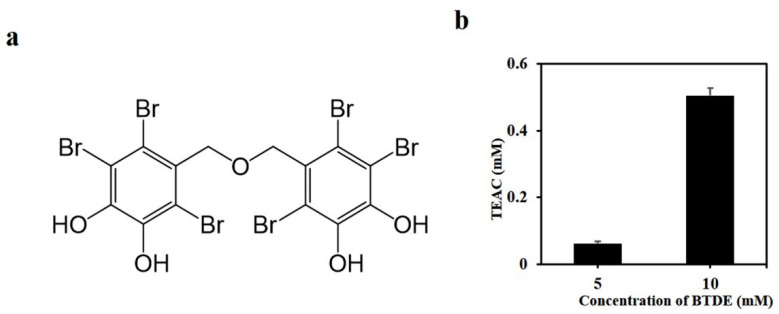
Bis (2,3,6-tribromo-4,5-dihydroxybenzyl) ether (BTDE) scavenges ABTS free radicals. (**a**) The chemical structure of BTDE. (**b**) The ability of BTDE to scavenge ABTS free radicals. BTDE was dissolved in DMSO and incubated with ABTS working solution, the absorbance value was detected at 734 nm, and the antioxidant capacity of BTDE was determined according to the standard curve. Values are expressed as mean ± SD of three independent experiments.

Bis (2,3,6-tribromo-4,5-dihydroxybenzyl) ether (BTDE, Figure 1a) is a typical representative molecule of natural BPs and was isolated from marine red alga *Symphyocladia latiuscula* [20]. Previous studies found that BTDE had antimicrobial [20], anti-diabetic [21], and anti-neurodegenerative activities [22]. In addition, BTDE was an inhibitor of tyrosinase [23] and glucose 6-phosphate dehydrogenase [24]. Interestingly, BTDE was also shown to effectively scavenge DPPH free radicals [25], indicating that it possesses antioxidant activity. However, BTDE’s antioxidant effect and protective function against skin oxidative damage have not been systemically studied, and there is much more about BTDE’s underlying antioxidant mechanisms that has yet to be elucidated.

Thus, in this study, we investigated both BTDE’s antioxidant effect and the corresponding molecular mechanisms. First, we confirmed BTDE’s antioxidant capacity in vitro using the ABTS^•+^ scavenging assay and assessed its cytoprotective effect on H_2_O_2_-induced damage in HaCaT cells. Then, we evaluated the effect of BTDE on the production of hydrogen peroxide (H_2_O_2_)-induced ROS, the malondialdehyde (MDA) level, the ratio of oxidized glutathione (GSSG)/glutathione (GSH), and the superoxide dismutase (SOD) activity in HaCaT cells. In addition, we examined whether BTDE could modulate Nrf2 and its controlled antioxidant proteins, as well as the possible signaling pathways involved in BTDE-stimulated Nrf2 activation.

## 2. Materials and Methods

### 2.1. Reagents and Drugs

BTDE (purity > 98%, amorphous light brown powder, dissolved in DMSO) was prepared by the School of Medicine and Pharmacy, Ocean University of China. Antibodies against HO-1, NQO1, TrxR1, AKT, phosphorylated AKT (S473), ERK1/2, phosphorylated ERK1/2 (T202/Y204), AMPKα, phosphorylated AMPKα, and Keap1 were purchased from Cell Signaling Technology (Boston, MA, USA). Antibody against GAPDH was obtained from Huaan Biotechnology Co., Ltd. (Hangzhou, China). Anti-Nrf2 antibody was obtained from Abcam Technology (Abcam Technology, Cambridge, UK).

### 2.2. Cell Lines and Cell Culture

The immortalized human keratinocyte cell line HaCaT and human umbilical vein endothelial cell line HUVEC were from American Type Culture Collection. Cells were maintained in dulbecco’s modified eagle’s medium (DMEM) (GIBCO, Grand Island, NY, USA) containing 10% fetal bovine serum (FBS) as well as 1% penicillin/streptomycin in a humidified incubator at 37 °C with 5% CO_2_.

### 2.3. ABTS^•+^ Scavenging Assay

The ABTS working solution was diluted with PBS buffer until the absorbance of the sample reached 0.7 ± 0.05 at 734 nm. Then, different concentrations of BTDE (5 and 10 mM) were incubated with ABTS solution, and the absorbance of the mixture was measured at 730 nm on a Microplate reader (BioTek, Winooski, VT, USA). Finally, the trolox equivalent antioxidant capacity (TEAC) value (mM) was calculated from the trolox standard curve.

### 2.4. Cell Viability Assessment

Cell viability was determined by sulforhodamine B (SRB) assay. Cells were seeded in 96-well plates and allowed to adhere overnight, then treated with different concentrations of BTDE (0–20 μM) for 24 h. For the H_2_O_2_ model, cells were treated with H_2_O_2_ (500 μM) for 3 h after pretreating with BTDE for 24 h. After the incubation was completed, cells were fixed with 10% trichloroacetic acid (100 μL) for 1 h at 4 °C. SRB (100 μL) was added and incubated for 15 min after rinsing and drying, and the bound stain was dissolved with Tris buffer (150 μL). Finally, a microplate reader (BioTek, Winooski, VT, USA) was used to measure the 96-well plate at 515 nm, and the cell viability (%) was calculated by OD value.

### 2.5. Cell Apoptosis Analysis

HaCaT cells were seeded in six-well plates (1 × 10^5^ cells/well) and allowed to adhere overnight. Then, cells were pretreated with BTDE (0–10 μM) for 24 h followed by H_2_O_2_ (500 μM) treatment for 3 h, and the control group was treated with dimethyl sulfoxide (DMSO). Cells were collected, centrifuged, and washed. Finally, cells were suspended in DMEM containing 1% FBS and stained with Muse^®^ Annexin V & Dead Cell Kit (Millipore, Billerica, MA, USA) for 20 min at 25 °C in the dark. After the staining, the proportions of apoptotic, necrotic, and living cells (%) were detected with Muse™ Cell Analyzer (Millipore, Billerica, MA, USA).

### 2.6. Intracellular ROS Assay

HaCaT cells were seeded in a 24-well plate treated with BTDE (5 and 10 μM) for 24 h. After the treatment, the cells were further incubated with 500 μM of H_2_O_2_ for 1 h. Subsequently, the cells were incubated with DCFH-DA (10 μM) in serum-free DMEM in the dark for 20 min. After the incubation, the cells were washed with serum-free DMEM, and the fluorescence intensity was detected by fluorescence microscopy (Leica, Germany). Finally, the ROS level was determined by fluorescence level.

### 2.7. Western Blotting Assay

HaCaT cells were seeded in six-well plates with a density of 1 × 10^5^ cells/well. Then, cells were treated with BTDE for an indicated time, and the control group was treated with DMSO. Cells were collected, washed, and lysed in loading buffer for 30 min at 4 °C. Then, the cell lysate was boiled for 10 min and stored at −20 °C. The protein samples were separated by electrophoresis on SDS-PAGE (6–12%) and transferred to nitrocellulose filter membranes (Millipore, Billerica, MA, USA). The nitrocellulose filter membranes were blocked with skimmed milk and then incubated with the primary antibodies. Subsequently, the membranes were incubated with HRP-secondary antibody at 25 °C for 1 h. Finally, the image was detected by Tanon 5200 (Tanon, Beijing, China).

### 2.8. Detection of Malondialdehyde (MDA), Superoxide Dismutase (SOD), and Oxidized Glutathione (GSSG)/Glutathione (GSH) Ratio

HaCaT cells were seeded in six-well plates with the density of 1.5 × 10^5^ cells/well overnight. The cells were treated with BTDE (5 and 10 μM) for 24 h and then incubated with H_2_O_2_ (500 μM) for 1 h. Then, the cells were collected, washed, and lysed in liquid nitrogen. Finally, the cellular levels of MDA, SOD and the GSSG/GSH ratio were measured using the commercially available kits, according to the manufacturer’s instructions (Beyotime, Shanghai, China).

### 2.9. Statistical Analysis

The results shown in this study were represented as the mean ± SD. Comparisons between the groups were assessed by one-way analysis of variance (ANOVA), and a multiple comparison test was performed using the post hoc Bonferroni correction. *p* < 0.05 was defined as statistically significant.

## 3. Results and Discussion

### 3.1. BTDE Scavenges ABTS Free Radicals

Previous investigations have found that BTDE had DPPH free radical scavenging activity [25]. In order to further confirm this antioxidant capacity, the ability of BTDE to scavenge ABTS free radicals was evaluated. The results showed that BTDE obviously scavenged in vitro ABTS free radicals (Figure 1b), suggesting its antioxidant activity.

### 3.2. BTDE Ameliorates H_2_O_2_-Induced Oxidative Cell Damage

**Figure 2:**
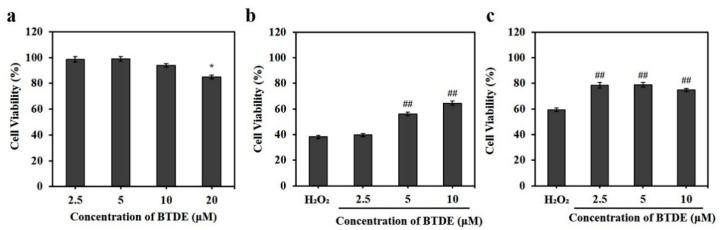
BTDE reverses oxidative damage in HaCaT and HUVEC cells. (**a**) The effect of BTDE on the viability of HaCaT cells. HaCaT cells were treated with different concentrations (2.5–20 μM) of BTDE for 24 h, and cell viability was then determined using the sulforhodamine B (SRB) method. (**b**) The effect of BTDE on the viability of HaCaT cells damaged by H_2_O_2_. HaCaT cells were incubated with BTDE for 24 h and then were treated with H_2_O_2_ for 3 h. Finally, cell viability was determined by the SRB method. (**c**) The effect of BTDE on the viability of HUVEC cells damaged by H_2_O_2_. HUVEC cells were incubated with BTDE for 24 h and then were treated with H_2_O_2_ for 3 h. Finally, cell viability was determined by the SRB method. Values are expressed as mean ± SD of three independent experiments. * *p* < 0.05, versus control group, ^##^ *p* < 0.01, versus H_2_O_2_ alone.

To further evaluate the antioxidant effect of BTDE at the cellular level, the keratinocyte cell line HaCaT, a cellular model widely used to investigate oxidative stress-induced skin diseases, was used to explore BTDE’s protective effect from oxidative stress. As shown in Figure 2a, BTDE could slightly inhibit the proliferation of HaCaT cells at high concentration (20 μM) after incubating for 24 h, but it could not at low concentrations (2.5–10 μM). Then, we used the non-toxic concentrations (2.5–10 μM) to investigate whether BTDE could ameliorate H_2_O_2_-induced oxidative cell damage. The results showed that BTDE (5–10 μM) could significantly reduce the cytotoxicity induced by H_2_O_2_ in HaCaT cells (Figure 2b). In HUVEC cells, BTDE (2.5–10 μM) could also reverse the oxidative damage induced by H_2_O_2_ (Figure 2c). Collectively, these results indicated that BTDE (5–10 μM) significantly attenuated H_2_O_2_-induced oxidative damage at the cellular level.

### 3.3. BTDE Ameliorates H_2_O_2_-Induced Cell Apoptosis

**Figure 3:**
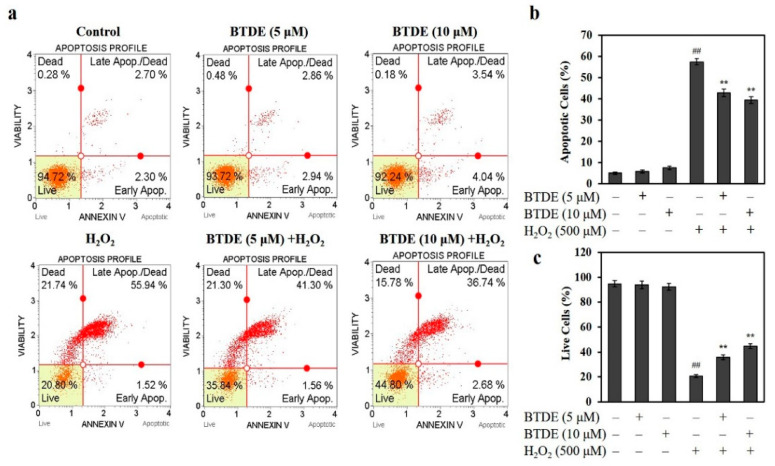
BTDE affects H_2_O_2_-induced HaCaT cell apoptosis and necrosis. (**a**) In the presence or absence of H_2_O_2_, the ratio of apoptosis of HaCaT cells treated with BTDE (5 and 10 μM) was measured with a MUSE cell analyzer. (**b**) The bar graph depicts the percentage of apoptotic HaCaT cells induced by H_2_O_2_ in the absence or presence of BTDE. (**c**) The bar graph depicts the percentage of surviving HaCaT cells induced by H_2_O_2_ in the absence or presence of BTDE. Values are expressed as the mean ± SD of three independent experiments. ^##^ *p* < 0.01, versus control group, ** *p* < 0.01, versus H_2_O_2_ alone.

To further evaluate the protective effect of BTDE on oxidative damaged cells, the effect of BTDE on H_2_O_2_-induced apoptosis of HaCaT cells was tested. As shown in Figure 3a,b, BTDE (5–10 μM) alone did not induce HaCaT cell apoptosis. H_2_O_2_ treatment increased the percentage of apoptotic cells to 57.46%. However, BTDE pretreatment significantly reduced the proportion of apoptotic cells to 42.86 and 39.42% at concentrations of 5 and 10 μM, respectively. Similarly, we also found that H_2_O_2_ treatment caused necrosis to 21.74% of cells (Figure 3a). However, the proportion of necrotic cells was significantly reduced to 15.78% after 10 μM BTDE pretreatment, with no significant difference between the 5 μM BTDE group and the H_2_O_2_ group (Figure 3a). Consistently, BTDE significantly increased the proportion of live cells, as shown in Figure 3a,c. Specifically, the proportion of living cells was 20.8% after H_2_O_2_ treatment, and after BTDE pretreatment at concentrations of 5 and 10 μM the proportion of living cells increased to 35.84 and 44.80%, respectively. Furthermore, in comparison with the percentage of living cells after treatment of 5 and 10 μM BTDE, we found that the higher concentration of BTDE showed a stronger effect, indicating that BTDE reduced H_2_O_2_-induced cell death and promoted cell survival in a concentration-dependent manner.

### 3.4. BTDE Alleviates H_2_O_2_-Induced ROS Generation, Decreased MDA Level and GSSG/GSH Ratio, and Increased SOD Activity

**Figure 4:**
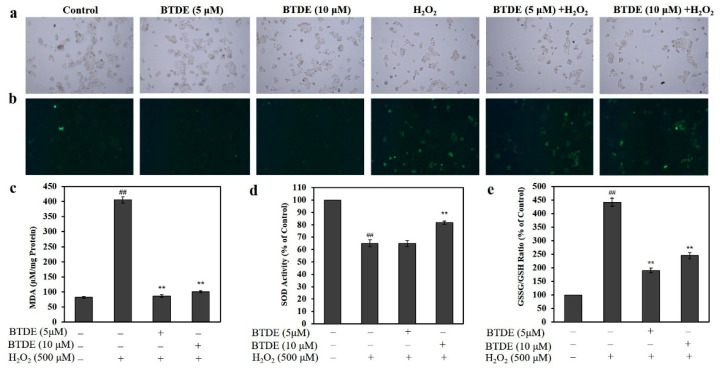
BTDE reduces ROS, MDA levels, and GSSG/GSH ratio, and it increases SOD activity in HaCaT cells. HaCaT cells were pretreated with BTDE (5 and 10 μM) for 24 h and then were exposed to H_2_O_2_ (500 μM) for 1 h. Cells were incubated with DCFH-DA (10 μM) for 20 min, and the cell morphology (**a**) and ROS level (**b**) were observed with fluorescence microscopy. The levels of MDA (**c**), SOD (**d**), and the ratio of GSSG/GSH (**e**) were measured using the indicated kit. Data are presented as the mean ± SD of three independent experiments. ^##^ *p* < 0.01, versus control group, ** *p* < 0.01, versus H_2_O_2_-treated group.

The excessive production of ROS is closely related to oxidative damage to the keratinocyte cells [26]. Therefore, we further assessed BTDE’s effect on the ROS level in HaCaT cells. As shown in Figure 4a,b, BTDE alone did not affect the level of ROS in HaCaT cells. H_2_O_2_ could significantly increase the production of ROS in HaCaT cells. However, BTDE could significantly reduce H_2_O_2_-induced ROS elevation in HaCaT cells (Figure 4a,b), and the decrease was more obvious at the concentration of 10 μM than at 5 μM, which indicated that BTDE could reduce ROS in a concentration-dependent manner. This result further confirmed that BTDE has antioxidant activity at the cellular level.

MDA is an indicator of lipid peroxidation [27]. After H_2_O_2_ treatment, the MDA level was significantly increased, while it decreased with BTDE pretreatment (Figure 4c). In living organisms, SOD is a natural superoxide free radical scavenger [28]. Our results found that BTDE pretreatment significantly increased the activity of SOD compared with H_2_O_2_ treatment alone (Figure 4d). Reduced glutathione (GSH) is an important antioxidant molecule for the maintenance of the organisms’ normal redox state. In the process of cellular defense against oxidative stress, the optimal balance between GSH and its oxidized form GSSG is quite important [29]. We found that BTDE could significantly decrease the ratio of GSSG/GSH (Figure 4e). However, BTDE had relatively weak effect on SOD activity as BTDE could not increase SOD activity at the concentration of 5 μM, while being able to decrease MDA level and GSSG/GSH at the same concentration, thus indicating that BTDE had different potency to modulate MDA level, SOD activity, and the ratio of GSSG/GSH, respectively. Overall, the above results suggested that BTDE alleviated H_2_O_2_-induced oxidative stress in HaCaT cells and had a stronger effect at the concentration of 10 μM.

### 3.5. BTDE Affects the Expression of Nrf2 and Keap1 in HaCaT Cells

**Figure 5:**
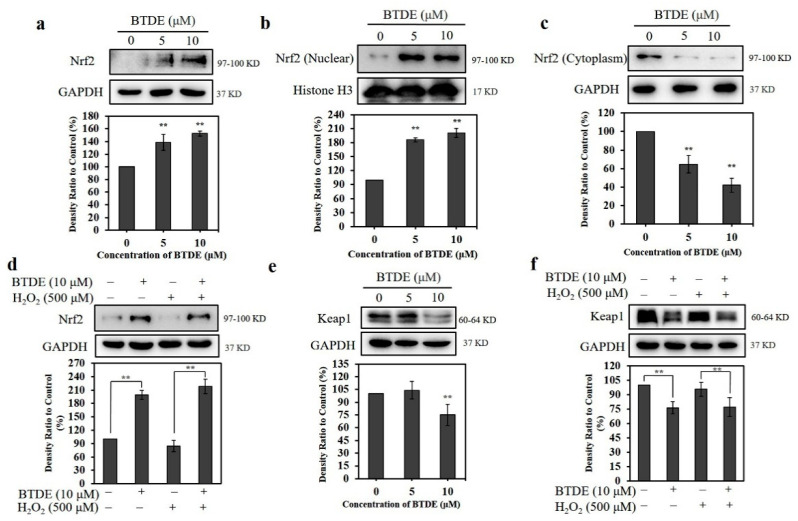
BTDE promotes the nuclear translocation of Nrf2 and decreases the expression of Keap1. (**a**) HaCaT cells were treated with BTDE (5–10 μM) for 24 h. The expression level of Nrf2 was detected by Western blotting assay. (**b**) HaCaT cells were treated with BTDE (5–10 μM) for 24 h. The nuclear expression level of Nrf2 was detected by Western blotting assay. (**c**) HaCaT cells were treated with BTDE (5–10 μM) for 24 h. The expression level of Nrf2 in cytoplasm was detected by Western blotting assay. (**d**) HaCaT cells were pretreated with BTDE (5–10 μM) for 24 h, and then incubated with or without H_2_O_2_ (500 μM) for 3 h. The expression level of Nrf2 was detected by Western blotting assay. (**e**) HaCaT cells were treated with BTDE (5–10 μM) for 24 h. The expression level of Keap1 was detected by Western blotting assay. (**f**) HaCaT cells were pretreated with BTDE (5–10 μM) for 24 h, and then incubated with or without H_2_O_2_ (500 μM) for 3 h. The expression level of Nrf2 was detected by Western blotting assay. Values are expressed as the mean ± SD of three independent experiments. ** *p* < 0.01, versus control group.

Since Nrf2 plays an important role in confronting cellular oxidative stress, we investigated the effect of BTDE on Nrf2 expression in HaCaT cells. As shown in Figure 5a, BTDE significantly increased the expression of Nrf2 in HaCaT cells in a concentration-dependent manner; furthermore, the Nrf2 in the nucleus increased (Figure 5b), while the Nrf2 in the cytoplasm decreased (Figure 5c), indicating that the nuclear translocation of Nrf2 also increased. Moreover, in the presence of H_2_O_2_ (which provides the oxidative stress condition) the expression of Nrf2 in HaCaT cells was increased significantly after BTDE pretreatment (Figure 5d), suggesting BTDE could increase the expression of Nrf2 under oxidative stress. These results indicate that BTDE could activate Nrf2 and counter oxidative stress in HaCaT cells by increasing Nrf2 expression and nuclear translocation.

Generally, Nrf2 is ubiquitinated and degraded through sequestration by Keap1 [30]. Therefore, we next investigated the effect of BTDE on the expression of Keap1 in HaCaT cells. As shown in Figure 5e, BTDE significantly decreased the expression of Keap1 in a concentration-dependent manner. Moreover, in the presence of H_2_O_2_ (which alone did not affect the Nrf2 or Keap1 level) under the present experimental conditions, the expression of Keap1 in HaCaT cells decreased significantly after BTDE pretreatment (Figure 5f). These results suggest that BTDE could increase Nrf2 expression and decrease the Keap1 level in HaCaT cells both in the presence and absence of exogenous oxidative stress. This finding was similar to previous studies exhibiting that 3-bromo-4,5-dihydroxybenzaldehyde attenuated H_2_O_2_-induced oxidative damage of HaCaT cells by increasing the expression of Nrf2 [17,18]. Moreover, the synthetic BP compound 1-(4-(4-bromo-2-(2-bromo-4,5-dihydroxybenzoyl)benzyl)piperazin-1-yl)ethan-1-one acted as an inhibitor of Keap1-Nrf2 co-interaction and had cytoprotective activity against H_2_O_2_-induced injury in EA.hy926 cells [19].

### 3.6. BTDE Increases the Expression of Nrf2-Mediated Proteins

**Figure 6:**
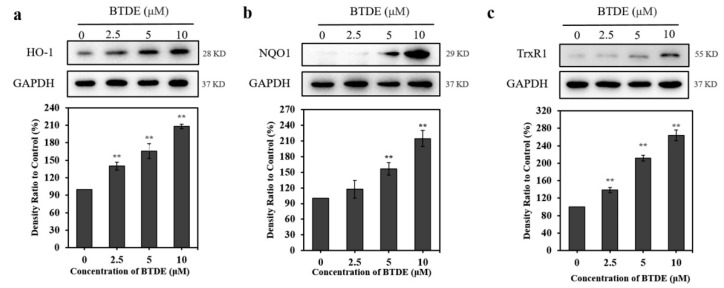
BTDE increases the expression of HO-1, NQO1, and TrxR1. HaCaT cells were treated with BTDE (2.5–10 μM) for 24 h. The expression levels of HO-1 (**a**), NQO1 (**b**), and TrxR1 (**c**) were detected by Western blotting assay. Values are expressed as the mean ± SD of three independent experiments. ** *p* < 0.01, versus control group.

Nrf2 promotes the expression of a variety of antioxidant proteins, such as HO-1, NQO1, and TrxR1, which all confront oxidative stress [31,32]. Thus, we next assessed the effect of BTDE on the expression of Nrf2 downstream antioxidant proteins in HaCaT cells. Our results, as shown in Figure 6, showed that BTDE significantly increased the expression of HO-1 (Figure 6a), NQO1 (Figure 6b), and TrxR1 (Figure 6c) in HaCaT cells in a concentration-dependent manner. Previous observation showed that bromophenol 3-bromo-4,5-dihydroxybenzaldehyde could increase the expression of both Nrf2 and HO-1 [18], and BTDE could also increase the expression of Nrf2 and Nrf2-mediated proteins, including HO-1, NQO1, and TrxR1. These results indicate that these BPs share a similar molecular mechanism which underlies their antioxidant capacity.

### 3.7. BTDE Activates the AKT Signaling Pathway

**Figure 7:**
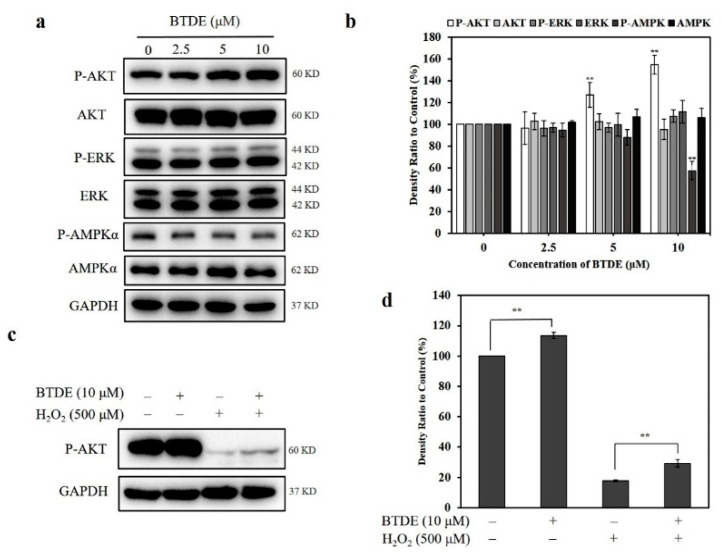
The effect of BTDE on the upstream signaling pathways of Nrf2. (**a**,**b**) HaCaT cells were treated with BTDE (2.5–10 μM) for 24 h. The expression levels of the signaling molecules were detected by Western blotting assay. (**c**,**d**) HaCaT cells were pretreated with BTDE (2.5–10 μM) for 24 h and then were incubated with or without H_2_O_2_ (500 μM) for 3 h. The expression level of P-AKT was detected by Western blotting assay. Values are expressed as the mean ± SD of three independent experiments. ** *p* < 0.01, versus control group.

It has been suggested that the AKT, ERK, and AMPK signaling pathways contribute to the activation of Nrf2 [17,33,34]. The activation of Nrf2 by these kinases as well as the decrease in Keap1 led to the nuclear translocation of Nrf2 and the expression of HO-1, NQO1, and TrxR1 [35]. Thus, we tested the effect of BTDE on the expression of the upstream signaling molecules of Nrf2 in HaCaT cells. As shown in Figure 7a,b, BTDE increased the expression of P-AKT in a concentration-dependent manner, while having no obvious effect on the activation of P-ERK and P-AMPKα. Furthermore, in the presence of H_2_O_2_, the expression of P-AKT in HaCaT cells was increased after BTDE pretreatment (Figure 7c,d). This result indicates that BTDE activated the AKT signaling pathway and contributed to the Nrf2 activation. The result was in accordance with previous findings demonstrating that BP 3-bromo-4,5-dihydroxybenzaldehyde increased Nrf2 expression through AKT phosphorylation in HaCaT cells [17,18]. However, while BTDE did not affect the ERK pathway, 3-bromo-4,5-dihydroxybenzaldehyde activated Nrf2 through both the ERK and the AKT pathways at the same time. This result shows that the way in which these two compounds mediated the activation of Nrf2 was not exactly the same, and this difference may be due to the different chemical structures of the two BPs.

## 4. Conclusions

In summary, for the first time, our present work demonstrated that BTDE effectively reduced oxidative stress-induced cellular damage by activating Nrf2. Specifically, we revealed that BTDE scavenged ABTS free radicals and ameliorated H_2_O_2_-induced HaCaT cell damage. Mechanism studies revealed that BTDE decreased H_2_O_2_-induced ROS generation, MDA level, and GSSG/GSH ratio, while it increased SOD activity. Most importantly, BTDE inhibited the expression of Keap1 and increased the expression of Nrf2 and its downstream proteins TrxR1, OH-1, and NQO1; furthermore, BTDE could activate the AKT signaling pathway, thus contributing to the activation of Nrf2. Although its in vivo antioxidant effects and the safety are unclear and need further investigation, this present study provides evidence that BTDE has strong antioxidant efficiency via modulating the Nrf2 pathway and thus could be developed as a new type of antioxidant for the treatment of skin damage as well as other diseases caused by oxidative stress.

## Data Availability

Data is contained within the article.
